# Lactate Dehydrogenase B and Pyruvate Oxidation Pathway Associated With Carfilzomib-Related Cardiotoxicity in Multiple Myeloma Patients: Result of a Multi-Omics Integrative Analysis

**DOI:** 10.3389/fcvm.2021.645122

**Published:** 2021-04-29

**Authors:** Marwa Tantawy, Lakshmi Manasa Chekka, Yimei Huang, Timothy J. Garrett, Sonal Singh, Chintan P. Shah, Robert F. Cornell, Rachid C. Baz, Michael G. Fradley, Nida Waheed, David L. DeRemer, Lihui Yuan, Taimour Langaee, Keith March, Carl J. Pepine, Jan S. Moreb, Yan Gong

**Affiliations:** ^1^Department of Pharmacotherapy and Translational Research, College of Pharmacy, University of Florida, Gainesville, FL, United States; ^2^Department of Pathology, Immunology and Laboratory Medicine, College of Medicine, University of Florida, Gainesville, FL, United States; ^3^Division of Hematology and Oncology, Department of Medicine, University of Florida, Gainesville, FL, United States; ^4^Division of Hematology and Oncology, Vanderbilt University Medical Center, Preston Research Building, Nashville, TN, United States; ^5^Department of Malignant Hematology, H. Lee Moffitt Cancer Center & Research Institute, Tampa, FL, United States; ^6^Cardio-Oncology Center of Excellence, Division of Cardiology, Department of Medicine, Perelman School of Medicine at the University of Pennsylvania, Philadelphia, PA, United States; ^7^Department of Internal Medicine, College of Medicine, University of Florida, Gainesville, FL, United States; ^8^UF Health Cancer Center, Gainesville, FL, United States; ^9^Department of Pharmacodynamics, College of Pharmacy, University of Florida, Gainesville, FL, United States; ^10^Center for Pharmacogenomics and Precision Medicine, College of Pharmacy, University of Florida, Gainesville, FL, United States; ^11^Division of Cardiovascular Medicine, Department of Medicine and Center for Regenerative Medicine, University of Florida, Gainesville, FL, United States; ^12^Novant Health Forsyth Medical Center, Hematology, Transplantation, and Cellular Therapy Division, Winston-Salem, NC, United States

**Keywords:** proteasome inhibitors, Cardio-oncology, carfilzomib, metabolomcis, proteomic

## Abstract

Multiple myeloma (MM) is the second most frequent hematologic cancer in the United States. Carfilzomib (CFZ), an irreversible proteasome inhibitor being used to treat relapsed and refractory MM, has been associated with cardiotoxicity, including heart failure. We hypothesized that a multi-omics approach integrating data from different omics would provide insights into the mechanisms of CFZ-related cardiovascular adverse events (CVAEs). Plasma samples were collected from 13 MM patients treated with CFZ (including 7 with CVAEs and 6 with no CVAEs) at the University of Florida Health Cancer Center. These samples were evaluated in global metabolomic profiling, global proteomic profiling, and microRNA (miRNA) profiling. Integrative pathway analysis was performed to identify genes and pathways differentially expressed between patients with and without CVAEs. The proteomics analysis identified the up-regulation of lactate dehydrogenase B (LDHB) [fold change (FC) = 8.2, *p* = 0.01] in patients who experienced CVAEs. The metabolomics analysis identified lower plasma abundance of pyruvate (FC = 0.16, *p* = 0.0004) and higher abundance of lactate (FC = 2.4, *p* = 0.0001) in patients with CVAEs. Differential expression analysis of miRNAs profiling identified mir-146b to be up-regulatein (FC = 14, *p* = 0.046) in patients with CVAE. Pathway analysis suggested that the pyruvate fermentation to lactate pathway is associated with CFZ-CVAEs. In this pilot multi-omics integrative analysis, we observed the down-regulation of pyruvate and up-regulation of LDHB among patients who experienced CVAEs, suggesting the importance of the pyruvate oxidation pathway associated with mitochondrial dysfunction. Validation and further investigation in a larger independent cohort are warranted to better understand the mechanisms of CFZ-CVAEs.

## Introduction

Multiple myeloma (MM) is a malignant plasma cell disorder and is the second most frequent hematologic cancer in the United States (1). Proteasome inhibitors (PIs) are among the most important classes of drugs to treat newly diagnosed, relapsed, and refractory MM (2). The mechanism of PI is dependent on the inhibition of proteasomal activity that disrupts the cell signaling pathways and leads to apoptosis and cell death (3, 4). So far, three PIs, bortezomib (Velcade®), carfilzomib (CFZ, Kyprolis®), and ixazomib (Ninlaro®), have been approved by the United States Food and Drug Administration (FDA) (5). CFZ is a second-generation PI that irreversibly inhibits 20S proteasome and is FDA-approved to treat relapsed or refractory MM patients who have received one to three previous treatments for MM. MM patients treated with CFZ and dexamethasone had improved survival compared with those treated with bortezomib and dexamethasone (6, 7).

Patients with MM often have cardiovascular diseases (CVDs) that may be a result of factors unrelated to MM, including older age, diabetes, obesity, or factors related to MM (such as amyloidosis), and other factors associated with the treatment of MM (such as anthracyclines, corticosteroids, alkylating agents, and PIs) (8). In an interim analysis of the phase 3 ENDEAVOR study, CFZ resulted in a significant and clinically meaningful survival benefit in relapsed MM patients compared with bortezomib, but it also was associated with significantly higher grade 3 cardiotoxicity, including heart failure (HF). In two meta-analyses, CFZ was associated with an increased incidence of cardiovascular adverse events (CVAEs) (8–18%) (2, 5) including hypertension, HF, arrhythmia, and cardiomyopathy (9, 10). CFZ accumulates in the heart, causes potent inhibition of the cardiac proteasome, and also decreases the 20S chymotrypsin-like activity to 10% in cardiomyocytes and 45% in bone marrow cells within 5 min after administration (11). Clinical trials and real-world experiences reported increasing incidence of CVAEs due to CFZ (CFZ-CVAE) in patients with MM compared with patients without MM with an adjusted hazard ratio (HR) ranging from 1.74 to 4.09 (8), which led to hospitalization, discontinuation of CFZ treatment, and also treatment-related death in ~5% of patients (12, 13). A critical clinical implication of this cardiotoxicity is treatment interruption that is associated with cancer recurrence (14). So far, there has been no human study investigating the mechanisms of CFZ-CVAE nor germline genetic risk factors for CFZ-CVAE. An integrative approach that combines multi-omics data, such as metabolomics, proteomics, transcriptomics, and microRNA (miRNA), may provide important insights into complex biological processes related to CFZ-CVAE.

This pilot study aimed to explore an integrative multi-omics approach to investigate data from different omics platforms including proteomics, metabolomics, and miRNAs to gain insights into the molecular mechanisms of CFZ-CVAE in MM patients.

## Materials and Methods

### Patients

The study was a retrospective cohort study of MM patients treated with CFZ at the University of Florida Health Cancer Center. Post-treatment whole blood samples were collected from MM patients who provided consent. CVAEs were evaluated in patients who had HF symptoms that led to obtaining the echocardiography and were confirmed by a cardiologist. A total of 13 MM patients were analyzed, including 7 who experienced CVAEs. This study was approved by the University of Florida Institutional Review Board (IRB) (IRB# 201702876).

### Global Metabolomic Profiling

Global metabolomic profiling of plasma samples was performed on a Thermo Q-Exactive Orbitrap mass spectrometer coupled with ultra-high-performance liquid chromatography (UHPLC) at the Southeast Center for Integrated Metabolomics at the University of Florida. Details of the analytical method are described in the method section of the Supplementary Materials. All metabolites from positive and negative ion modes were separately subjected to quality control steps, data normalization, and statistical analyses using MetaboAnalyst 4.0 (15, 16). *T*-test and partial least square discrimination analysis (PLS-DA) were performed to identify metabolites with different abundance in those with and without CVAEs. Known metabolites with fold change (FC) of >1.5 and raw *p*-value of <0.001 were selected for pathway analysis using Ingenuity Pathway Analysis (IPA) (Qiagen IPA, USA).

### Global Proteomics Profiling

Global proteomic profiling was performed on the plasma samples using the isobaric tags for relative and absolute quantification (iTRAQ) high-throughput proteomic method on these 13 MM patients treated with CFZ to identify proteomic biomarkers associated with CFZ-CVAEs. For quantification, only MS/MS spectra that were unique to a particular peptide and the sum of the signal-to-noise ratios for all the peak pairs >9 were used. The ratios with *p* < 0.05 present in at least two replicates were considered significant. Only the significant ratios from the replicates were used to calculate the average ratio for the protein. Details of the analytical method are described in the Method section of the Supplementary Materials.

### Circulating miRNA Extraction and Profiling

Total RNA was extracted from the plasma samples using the miRVana microRNA Isolation Kit (Thermo Fisher Scientific, USA) and reverse transcribed using Megaplex™ RT Primers. Preamplification of cDNA with Megaplex™ PreAmp primers was performed according to the manufacturer's protocol and recommendations (Thermo Fisher Scientific, USA). miRNA expression was quantified using TaqMan® OpenArray® Human MicroRNA Panel containing 754 human miRNAs in QuantStudio™ 12K Flex Real-Time PCR.

### Statistical Analysis

The characteristics of the patients were summarized with mean and standard deviation, median and range, or number and percentages as appropriate. The characteristics of patients with and without CVAEs were compared using Fisher's exact test for categorical variables and Mann–Whitney U test for continuous variables.

The high-throughput data generated from TaqMan® OpenArray® RT-qPCR, the miRNAs with Cq-values above 35, and the amplification score below 1.24 were excluded. The combination of miR-24 and miR-16 was used for normalization according to the NormFinder software suggestion with the most stable value of 0.005 (17). MiRNAs were determined to be significantly up- or down-regulated in MM samples with CVAEs and FC >1.5 or <0.5, *p* ≤ 0.05; the 2^−ΔΔCT^ method was used for analysis (18). All statistical analyses were performed using SAS v.9.4 (Cary, NC, USA), Statistical Package for the Social Sciences SPSS v.26 (IBM Corp., USA), or R software.

### Multi-Omics Integrative Analysis and Prediction of Biological Functions and Networks

The canonical pathways and networks associated with the differentially expressed metabolites, proteins, and miRNAs were identified using Qiagen's Ingenuity® Pathway Analysis version 6.0 (IPA® QIAGEN Bioinformatics, CA, USA; www.qiagen.com/ingenuity). IPA helps to predict the biological activity across thousands of datasets that represent disease conditions. By incorporating the top proteins, metabolites, and miRNAs, the canonical pathways associated with the multi-omics data can be identified. The pathways enriched with the top signals in any of the multi-omics data sources were identified using Fisher's exact test.

## Results

A total of 13 MM patients treated with CFZ were analyzed, including 7 (53.8%) who experienced CVAEs. The overall median age was 58 years (range 46–76), and 38.5% were men. The characteristics of patients with or without CVAEs are summarized in Table 1. There were no statistical differences in demographics, comorbidities, or MM status between the patients who developed CVAEs and those who did not. There was no significant difference in baseline left ventricular ejection fraction (LVEF) between the two groups. However, there was a trend of lower LVEF in patients with CVAE (median 32.5) than in those without CVAEs (median of 57.5) (*p* = 0.08).

**Table 1:** Demographic and clinical characteristics of patients treated with CFZ.

	**CVAE (*n* = 7)**	**No CVAE (*n* = 6)**	***p*-value**
Age, years (mean ± SD)	64 ± 9.4	55.5 ± 9.7	0.22
Male sex,	2 (28.5%)	3 (50%)	0.59
Diabetes	2 (28.5%)	0	0.46
Hypertension	3 (43%)	6 (100%)	0.07
Hyperlipidemia	2 (28.5%)	2 (33.4%)	0.99
Chronic kidney disease	2 (28.5%)	2 (33.4%)	0.99
Baseline LVEF, median (minimum; maximum)	55 (50–65)	62.5 (55–65)	0.44
Follow-up LVEF, median (minimum; maximum)	32.5 (20–60)	57.5 (50–67.5)	0.08
**MM status**			
Complete remission	3 (43%)	2 (33.5%)	0.99
Partial remission	1 (14%)	1 (16.5%)	
Relapsed/progressive disease	3 (43%)	3 (50%)	

*CVAE, cardiovascular adverse event; SD, standard deviation; LVEF, left ventricular ejection fraction; MM, multiple myeloma*.

*Categorical variables are summarized as n (%)*.

*p-values were from Fisher's exact test for categorical variables and Mann–Whitney U test for continuous variables*.

### Metabolomic Profiling

**Figure 1:**
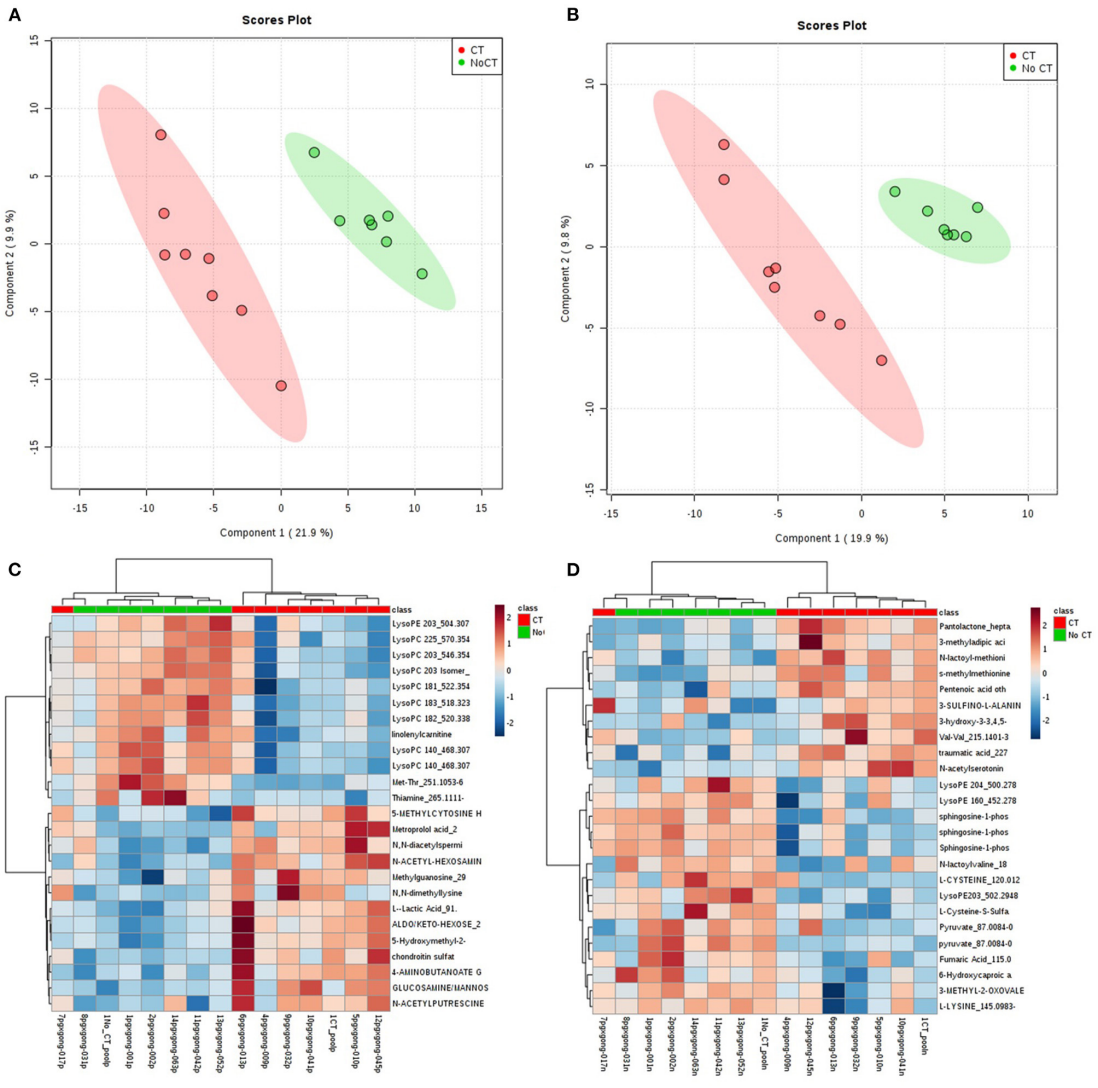
Metabolomic profiling of MM patients treated with CFZ. Green color: patients without CVAE. Red color: patients with CVAE. **(A)** PLS-DA 2D scores plot of metabolites in the positive ion channel; **(B)** PLS-DA 2D scores plot of metabolites in the negative ion channel; **(C)** heatmap of the top 25 metabolites in the positive ion channel; **(D)** heatmap of the top 25 metabolites in the negative ion channel.

**Figure 2:**
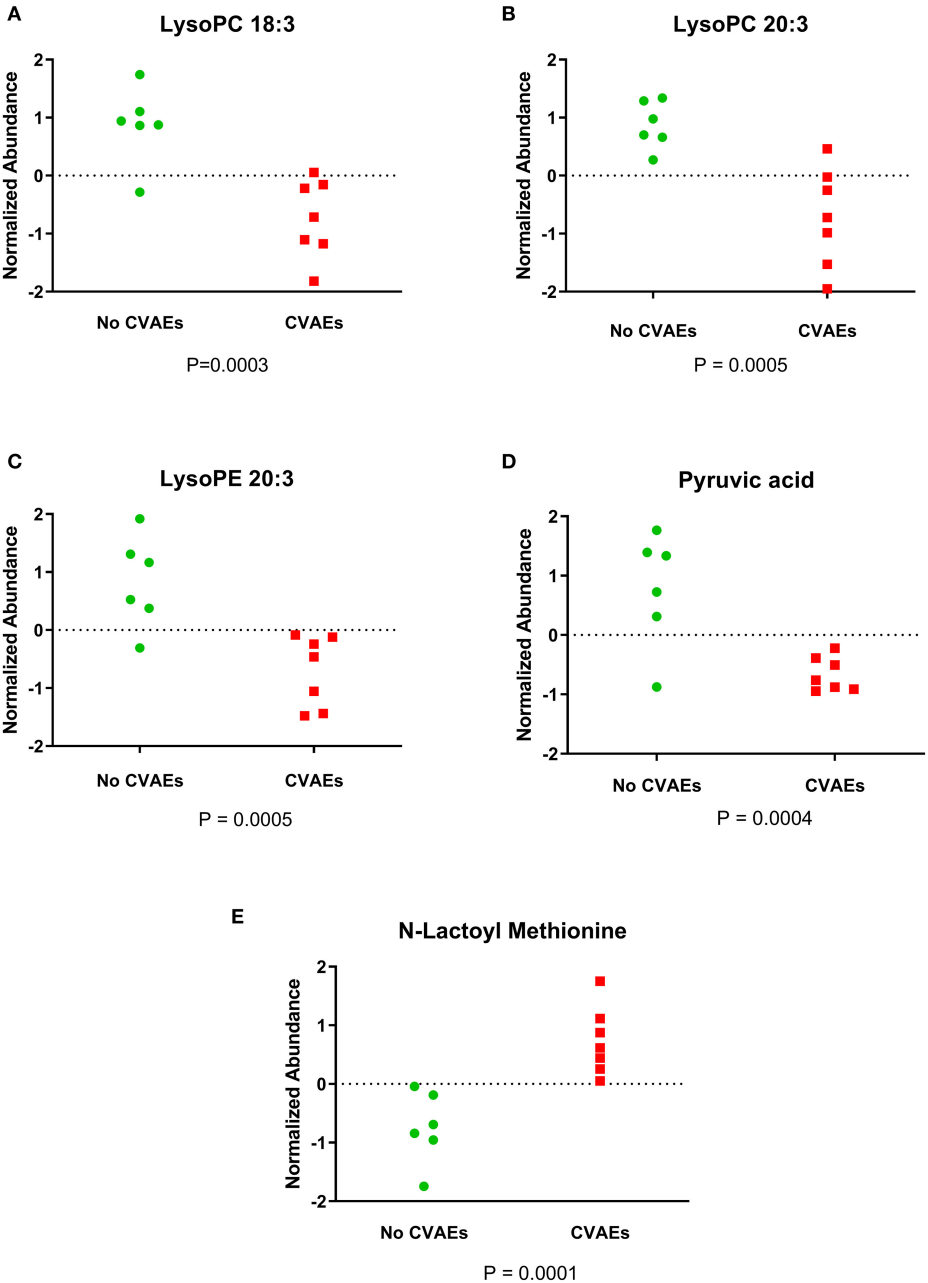
Figure showing the normalized abundance of metabolites with significantly different levels between patients with and without CVAEs after treatment with CFZ. **(A)** LysoPC 18:3, **(B)** LysoPC 20:3, **(C)** LysoPE 20:3, **(D)** pyruvic acid, **(E)** N-lactoyl methionine.

A total of 8,636 metabolites were identified in the untargeted metabolomic profiling. After quality control, a total of 375 known metabolites were analyzed. The PLS-DA scores plots indicated a good separation of metabolites between patients with and without CVAEs (Figures 1A,B). Seventeen metabolites had FC >1.5 and raw *p* < 0.001. The top 25 metabolites that differentiated these two groups of patients in positive and negative ionization are illustrated in the heatmaps (Figures 1C,D). Among the top metabolites, lower intensities of lysophospholipids were identified in patients with CVAEs compared with patients without CVAEs. For example, the abundance of lysophosphatidylcholines [LysoPC (18:3), HMDB0010387] in patients with CVAEs was 33% of that in patients without CVAEs (*p* = 0.0003). The abundance of LysoPC (20:3, HMDB0010393) in patients with CVAEs was 39% of that in patients without CVAEs (*p* = 0.0005). The abundance of lysophosphatidylethanolamine [LysoPE (20:3), HMDB0011515] in patients with CVAEs was 44% of that as compared with patients without CVAEs (*p* = 0.0005) (Figures 2A–C).

Additionally, the metabolomics data analysis identified lower abundance of pyruvic acid (HMDB0000243) in patients with CVAEs as 16% of that seen in patients without CVAEs (*p* = 0.0004), but an increased abundance of the lactate modified amino acid (N-lactoyl-methionine, HMDB0062182) (2.4-fold) in patients with CVAEs compared with the abundance in patients without CVAEs (*p* = 0.0001) (Figures 2D,E). All top metabolites that differentiated patients with and without CVAEs (with FC >1.5 or <0.5, *p* < 0.01) are listed in Table 2.

**Table 2:** Top metabolites that differentiate patients with and without CVAEs (with fold change >1.5 or <0.5 and *p* < 0.01).

**HMDB ID**	**Metabolite name**	**Fold change**	***p*-value**
HMDB0028983	Methionyl-threonine	0.19	0.00008
HMDB0062182	N-lactoyl-methionine	2.38	0.00014
HMDB0010387	LysoPC (18:3(6Z,9Z,12Z)/0:0)	0.33	0.00032
HMDB0000243	Pyruvic acid	0.16	0.00049
HMDB0010393	LysoPC (20:3(5Z,8Z,11Z)/0:0)	0.39	0.00053
HMDB0011515	LysoPE (20:3(5Z,8Z,11Z)/0:0)	0.44	0.00055
HMDB0002894	5-Methylcytosine	1.97	0.00085
HMDB0000277	Sphingosine-1-phosphate	0.31	0.00133
HMDB0041947	N1, N8-Diacetylspermidine	2.29	0.00144
HMDB0032642	5-Methyl-5-pentacosanol	2.70	0.00145
HMDB0000933	Traumatic acid	1.98	0.00163
HMDB0010379	LysoPC (14:0/0:0)	0.37	0.00188
HMDB0010386	LysoPC (18:2(9Z,12Z)/0:0)	0.38	0.00263
HMDB0038670	S-Methylmethionine	3.93	0.00273
HMDB0000190	⌞-Lactic acid	1.68	0.00303
HMDB0000574	⌞-Cysteine	0.21	0.00310
HMDB0001563	1-Methylguanosine	2.16	0.00368
HMDB0010402	LysoPC(22:5(4Z,7Z,10Z,13Z,16Z)/0:0)	0.49	0.00423
HMDB0000112	gamma-Aminobutyric acid	3.17	0.00447
HMDB0034355	5-Hydroxymethyl-2-furancarboxaldehyde	1.54	0.00462
HMDB0006469	Linoleyl carnitine	0.28	0.00477
HMDB0000235	Thiamine	0.12	0.00539
HMDB0002815	LysoPC (18:1(9Z)/0:0)	0.39	0.00580
HMDB0013287	Ne, Ne dimethyllysine	2.88	0.00606
HMDB0001434	3-Methoxytyrosine	4.00	0.00608
HMDB0001276	N1-Acetylspermidine	3.38	0.00676
HMDB0010392	LysoPC (20:2(11Z,14Z)/0:0)	0.42	0.00724
HMDB0032459	2-Pentenoic acid	2.38	0.00734
HMDB0094704	N-Propionylmethionine	4.43	0.00758
HMDB0011512	LysoPE (20:1(11Z)/0:0)	0.39	0.00839
HMDB0011502	LysoPE (15:0/0:0)	0.39	0.00863
HMDB0059876	Pantolactone	7.38	0.00869
HMDB0010384	LysoPC (18:0/0:0)	0.40	0.00911
HMDB0062181	N-Lactoylvaline	0.40	0.00930
HMDB0000195	Inosine	2.37	0.00973
HMDB0000070	Pipecolic acid	0.22	0.01056

### Proteomic Profiling

**Figure 3:**
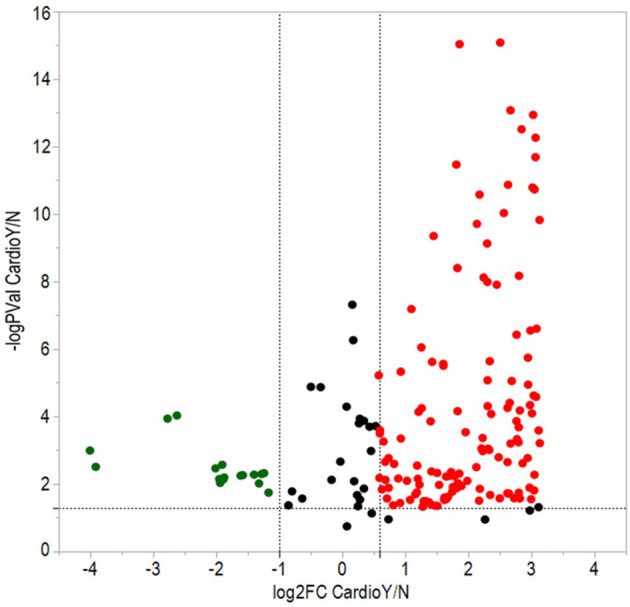
Volcano plot of differentially expressed proteins between patients with and without CVAEs after treatment with CFZ. Negative log_10_ *p*-values were plotted on the y-axis, and log_2_ normalized fold changes expression levels on the x-axis. Significant differential expression with *p*-value threshold values (α = 0.05) was detected at <0.5 or >1.5-fold changes. Red and green dots indicate up- and down-regulated proteins in patients with CVAEs relative to patients without CVAEs.

From a total of 374 identified proteins, 347 were quantified with 202 excluded due to variation within the non-CVAE group. Fifty-seven proteins were differentially expressed, including 12 proteins down-regulated and 45 up-regulated in MM patients with CVAEs compared with those without CVAEs (FC >1.5 or <0.5, *p* < 0.05) (Table 3). The differential expression between the two groups is presented in the volcano plot (Figure 3). The top proteins that were up-regulated in patients with CVAEs compared with those without CVAEs include complement factor D (CFD) (FC = 6.15, *p* = 0.0006), lactate dehydrogenase B (LDHB) (FC = 8.24, *p* = 0.015), and insulin-like growth factor-binding protein 2 (IGFBP2) (FC = 7.67, *p* = 0.0017) (Table 3).

**Table 3:** The list of a total of 57 differentially expressed proteins at <0.5 or >1.5 of fold changes with *p* < 0.05.

**UniProt ID**	**Protein name**	**Fold change**	***p*-value**
F8WA32	Testis-specific gene 10 protein (Fragment)	12.69	0.00001
K7ERG9	Complement factor D	6.15	0.00006
H0YIS8	Transmembrane protein 19 (Fragment)	0.15	0.00012
P05543	Thyroxine-binding globulin	9.90	0.00027
P18065	Insulin-like growth factor-binding protein 2	7.67	0.00169
Q9Y5Z9	UbiA prenyltransferase domain-containing protein 1	1.66	0.00169
Q8NDI1	EH domain-binding protein 1	1.77	0.00252
B3KT58	cDNA FLJ37685 fis, clone BRHIP2013972	0.27	0.00268
Q96L14	Cep170-like protein	0.07	0.00308
A0A087WZT2	Methyltransferase-like protein 7B	0.25	0.00341
A6NKC6	Retinitis pigmenta 1-like 1 protein	2.84	0.00465
Q9Y2Z9	Ubiquinone biosynthesis monooxygenase COQ6, mitochondrial	0.42	0.00473
Q6PIQ7	IGL@ protein	3.62	0.00489
O60662	Kelch-like protein 41	0.41	0.00504
A0A087WWH3	BRD4-interacting chromatin-remodeling complex-associated protein	0.38	0.00524
A0A1W2PPP1	Gamma-aminobutyric acid receptor subunit delta (Fragment)	0.33	0.00543
Q8IV28	NID2 protein	0.33	0.00562
Q8NCL6	cDNA FLJ90170 fis, clone MAMMA1000370, highly similar to Ig alpha-1 chain C region	3.13	0.00595
O75882	Attractin	1.85	0.00683
J3KSY5	Hydrocephalus-inducing protein homolog (Fragment)	0.26	0.00707
A0A0K0K1J1	Cystatin	1.60	0.00751
Q60FE6	Filamin A	2.04	0.00798
P02452	Collagen alpha-1(I) chain	3.95	0.00802
Q5W0U4	B box and SPRY domain-containing protein	35.76	0.00819
Q5JRA6	Transport and Golgi organization protein 1 homolog	0.26	0.00925
L8E853	von Willebrand factor	3.56	0.00940
O60503	Adenylate cyclase type 9	0.40	0.00956
P11226	Manne-binding protein C	2.34	0.01042
P61769	Beta-2-microglobulin	20.23	0.01124
A0A087WZA1	HCG1820835, isoform CRA_a	1.66	0.01289
Q53H91	Phospholipid transfer protein isoform a variant (Fragment)	1.55	0.01435
Q5U077	l-Lactate dehydrogenase	8.24	0.01538
A0A0D9SFL2	Bifunctional polynucleotide phosphatase/kinase	3.35	0.01685
F1C4A7	Monocyte differentiation antigen CD14	2.27	0.01759
B4DPQ3	cDNA FLJ51034, highly similar to Vitamin K-dependent protein C (EC 3.4.21.69)	6.14	0.01869
H0Y2W4	Protein MROH8 (Fragment)	5.08	0.02101
V9HWE3	Carbonic anhydrase I, isoform CRA_a	14.59	0.02330
Q6ZMG9	Ceramide synthase 6	3.20	0.02376
A0A024R6H0	Cleavage and polyadenylation specific factor 2, 100 kDa, isoform CRA_a	3.07	0.02501
C9J6K0	Secreted phosphoprotein 24 (Fragment)	6.61	0.02618
A0A024RAG6	Complement component 1, q subcomponent, A chain, isoform CRA_a	5.65	0.02649
Q7Z379	Uncharacterized protein DKFZp686K04218 (Fragment)	1.63	0.02666
Q86TT1	Full-length cDNA clone CS0DD006YL02 of Neuroblastoma of *Homo sapiens* (human)	3.14	0.02779
Q6IMJ8	NOELIN1_V1	7.97	0.02800
B1AHL2	Fibulin-1	2.11	0.02931
A2J422	Anti-HER3 scFv (Fragment)	3.07	0.02943
O00391	Sulfhydryl oxidase 1	4.50	0.03095
P23142	Fibulin-1	2.45	0.03207
Q15075	Early endome antigen 1	2.50	0.03218
Q6FGQ2	PHYH protein (Fragment)	2.59	0.03423
A0A1B0GU74	Voltage-dependent P/Q-type calcium channel subunit alpha	2.65	0.04073
A0A125QYY5	GCT-A9 light chain variable region (Fragment)	8.92	0.04191
Q53GU8	Transforming growth factor, beta-induced, 68 kDa variant (Fragment)	1.75	0.04238
Q96QS0	Putative matrix cell adhesion molecule-3	2.78	0.04372
H0YD13	CD44 antigen	2.84	0.04423
A0A024R3C7	Ataxia telangiectasia mutated (Includes complementation groups A, C and D), isoform CRA_a	2.42	0.04672
Q13790	Apolipoprotein F	8.66	0.04842

### MiRNA Profiling

**Figure 4:**
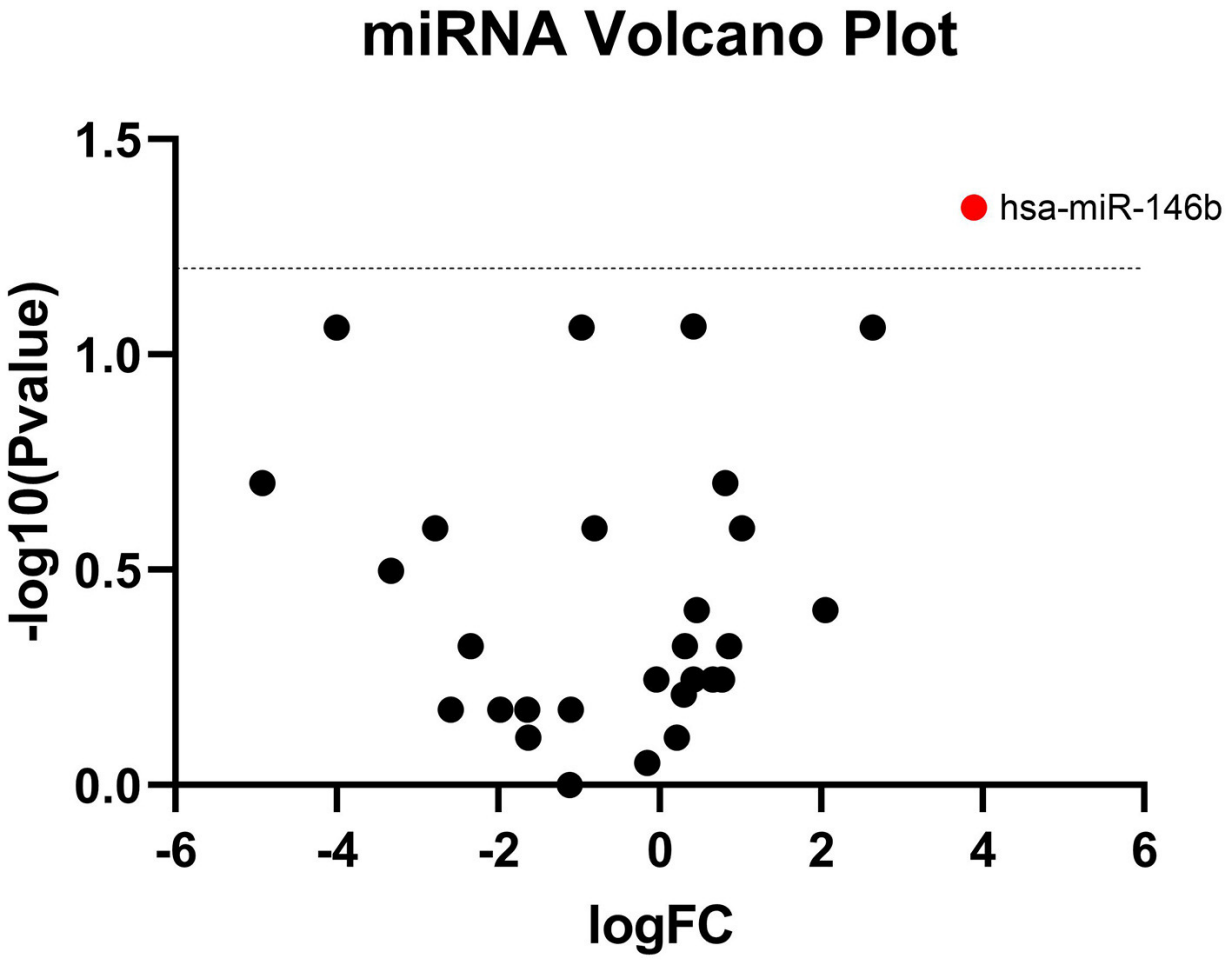
Volcano plot of differential expression of miRNA between patients with and without CVAEs. MicroRNAs are differentially expressed in MM patients treated with CFZ. Volcano plot of significantly up-regulated (red dots) and down-regulated (green dots) microRNAs in patients with and without CVAEs as detected by TaqMan OpenArray profiling. Negative log_10_ *p*-values were plotted on the y-axis, and log_2_ normalized fold changes expression levels on the x-axis. A significant differential expression was detected with the *p* ≤ 0.05.

TaqMan OpenArray analyzed a total of 750 miRNAs in 13 MM patients treated with CFZ. Twenty-one of these miRNAs were identified to be up-regulated or down-regulated in patients with CVAEs compared with patients without CVAEs (Table 4). Figure 4 shows the volcano plot of the OpenArray miRNAs data that were up-regulated or down-regulated in MM patients with CVAEs compared with those without CVAEs. Differential expression analysis of miRNAs profiling identified mir-146b to be up-regulated in those with CVAEs compared with those without CVAEs (FC = 14, *p* = 0.046).

**Table 4:** MicroRNA up-regulated or down-regulated in MM with or without CVAEs as determined by Open Array fold change <0.5 and ≥1.5.

**miRNA name**	**Z score**	***p*-value**	**Fold change**	**Regulation**
hsa-miR-146b	−2.00	0.046	14.80	Up
hsa-miR-342-3p	−1.71	0.086	6.22	Up
hsa-miR-19b	−1.71	0.086	0.51	Down
hsa-miR-30b	−1.71	0.086	0.06	Down
hsa-miR-1274B	−1.29	0.199	1.76	Up
hsa-miR-139-5p	−1.29	0.199	0.03	Down
hsa-miR-150	−1.14	0.253	2.02	Up
hsa-miR-17	−1.14	0.253	0.57	Down
hsa-miR-195	−1.14	0.253	0.15	Down
hsa-miR-30a-5p	−1.00	0.317	0.10	Down
hsa-miR-191	−0.86	0.391	4.15	Up
hsa-miR-331	−0.71	0.475	1.81	Up
hsa-miR-142-3p	−0.71	0.475	0.20	Down
hsa-miR-222	−0.57	0.568	1.70	Up
hsa-miR-126	−0.57	0.568	1.57	Up
hsa-miR-30c	−0.43	0.668	0.47	Down
hsa-miR-106a	−0.43	0.668	0.32	Down
hsa-miR-720	−0.43	0.668	0.25	Down
hsa-miR-483-5p	−0.43	0.668	0.17	Down
hsa-miR-21	−0.29	0.775	0.32	Down
hsa-miR-92a	0.00	1.000	0.46	Down

### Pathway Analysis Using IPA

The top proteins and metabolites that were differentially expressed in the two groups were incorporated into the IPA analysis. The top two most significant canonical pathways associated with CFZ-CVAEs are the glutamate-dependent acid resistance pathway (*p* = 0.006) and pyruvate fermentation to lactate pathway (*p* = 0.01).

## Discussion

In this pilot study, we performed integrative multi-omics analyses of metabolomic, proteomic, and miRNA data to identify differences among MM patients with or without CVAEs. Up-regulation of LDHB, lower plasma abundance of pyruvate, higher plasma abundance of lactate, and up-regulation of mir-146b were observed in patients with CVAEs compared with those without CVAEs. The most significant pathway identified was the glutamate-dependent acid resistance pathway that is related to fatty acid biotransformation activity. (19) The second top significant pathway, the pyruvate oxidation pathway, was related to mitochondrial dysfunction (20).

Mitochondria is the active organelle the produces most reactive oxygen species (ROS) that leads to peroxidation of lipids and DNA damage in cardiomyocytes (21–23). In cardiac cells, the LDHB converts lactate to pyruvate, which is considered the critical metabolite in glycolysis and glucose oxidation. The pyruvate oxidation (pyruvate decarboxylation) occurs after pyruvate is converted to acetyl-CoA in the mitochondria by the pyruvate dehydrogenase complex (PDC). Acetyl-CoA then is used in the citric acid cycle (CAC) for adenosine triphosphate (ATP) production, which is essential for cardiomyocyte contractility (24). The decarboxylation of pyruvate is dominant in the mitochondria of healthy cardiac cells (24). Mitochondrial protein complex (MPC) is a mitochondrial inner-protein complex that consists of two subunits: MPC1 and MPC2. Both subunits are essential for the normal function of MPC, and loss of any subunit would affect the mitochondrial pyruvate transport and the pyruvate supporting the oxygen consumption in cardiac cells (25). A recent study identified a correlation between LDHB and cardiac hypertrophy and suggested a molecular mechanism for regulating abnormal energy metabolism in cardiac hypertrophy (26–28). Based on our preliminary results, there was compelling evidence for the pyruvate oxidation pathway's involvement in CFZ-CVAEs.

A previous study of mice published by Efentakis and colleagues suggested that CFZ induces cardiotoxicity through increased protein phosphatase 2 (PP2A) activity and inhibition of AMP-activated protein kinase (AMPK) and its downstream autophagic targets (4). Our untargeted metabolomic analyses identified significantly lower abundance of phospholipids, such as LysoPC. In another study in non-cancer patients with Heart Failure Reduced Ejection Fraction (HFrEF), the authors also found decreasing phospholipids compared with healthy controls matched by age, sex, body mass index, and ischemic heart disease status (29). Lower lysophospholipid levels coupled with higher long-chain acylcarnitine levels may reflect a shift toward increased myocardial glucose oxidation, with down-regulation of fatty acid oxidation as the result of mitochondrial dysfunction in HF patients. Lower lysophospholipid levels result from increased PP2A activity, which is an essential and ubiquitously expressed serine–threonine phosphatase, plays a critical role in cellular processes, such as cell proliferation and apoptosis, and is responsible for more than 90% of protein dephosphorylation in cardiac cells (30, 31). Studies have shown that lower levels of lysophospholipids are likely to increase PP2A activity (32) and inhibition of AMPK (33). Our findings of metabolomic readout of MM patients treated with CFZ were consistent with a previously published study in mice.

We also discovered the CFD to be significantly up-regulated in patients with CVAEs. CFD is targeted by miR-17, miR-19b, miR-92a, miR-30b,c, and miR-106a (34). CFD also promotes the alternative pathway complement (AP) activation, and the complement activation was shown to occur in chronic HF and is associated with adverse clinical events (35). These findings suggest that CFZ may induce cardiac dysfunction *via* increasing CFD activity, leading to the complement system's activation. We also observed an elevation of IGFBP2 in patients who experienced CVAEs. Previous studies reported that IGFBP2 might play an essential role as a potential diagnostic and prognostic marker for suspected HF (30, 36, 37). However, there is still a lack of information about the structure and function of IGFBPs associated with CVD.

In our study, we also detected the up-regulation of miR-146b in patients with CVAEs. In a study performed on rats, the authors reported the role of miR-146b during myocardial ischemia/reperfusion injury (IRI) in myocardial I/R rat and hypoxia/reoxygenation (H/R) injured cardiomyocytes. They found that miR-146b overexpression significantly decreased the myocardial infarct size and cardiomyocytes apoptotic rates and release of creatine kinase and lactate dehydrogenase during IRI by targeting Smad4 and modulating the expression of c-fos and c-JUN in the myocardium (31). Another study showed that miR-146b inhibition increased hypoxia-induced cardiomyocyte apoptosis, which indicated the potential protective effect of miR-146b in ischemia injury (38). However, the role of miR-146b in myocardial ischemic injury is still unclear (4).

A healthy heart relies primarily on fatty acid oxidation for ATP production, with a smaller contribution from glucose oxidation. In early HF, glucose oxidation increases, and glucose is converted to pyruvate in the cytosol, then transported into the mitochondria, and converted to acetyl Co-A by pyruvate dehydrogenase for ATP production and to lactate by LDHB. Both fatty acid and glucose oxidation are decreased in advanced HF (39–41). In this cohort study, proteomics analysis identified the up-regulation of LDHB in patients who experienced CVAEs. On the other hand, the metabolomics data analysis showed lower level of pyruvate but increased level of lactate in patients with CVAEs. A recent study identified a correlation between LDHB and cardiac hypertrophy and suggested a molecular mechanism for regulating abnormal energy metabolism in cardiac hypertrophy (26). LDHB may be an appropriate target to improve abnormal cardiac energy metabolism in the setting of cardiac hypertrophy.

**Figure 5:**
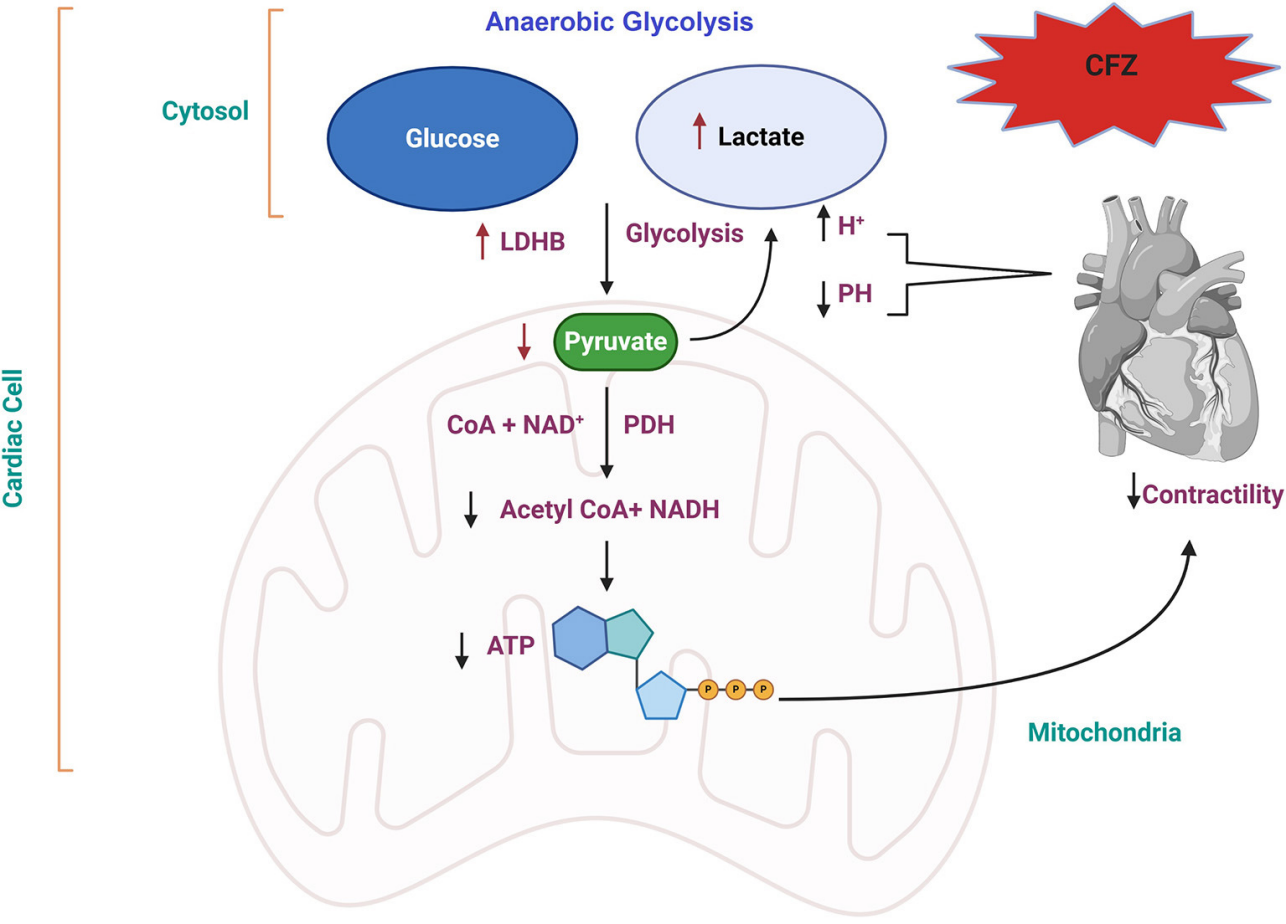
Proposed molecular mechanism of CFZ-CVAEs. Red arrows indicate the changes in this study. CFZ, carfilzomib; LDHB, lactate dehydrogenase B; PDH, pyruvate dehydrogenase; NADH, nicotinamide adenine dinucleotide hydrogen; ATP, adenosine triphosphate.

Based on the integrative analysis of multi-omics data, we identified the association between the pyruvate fermentation to lactate (pyruvate oxidation) pathway and CFZ-CVAEs. Our results highlight the increase in lactate level and decrease of pyruvate level after CFZ administration, which supports the idea that anaerobic glycolysis occurring in myocardial ischemia leads to mitochondrial metabolic dysfunction and reduction in ATP production through oxidative phosphorylation (42, 43). We propose a possible mechanism for the CFZ-CVAEs in Figure 5. A lower level of pyruvate and a higher level of lactate in patients with CVAEs are consistent with cardiomyocytes switching substrate preference from fatty acids to glucose because of mitochondrial dysfunction.

It is important to acknowledge a couple of limitations of our study. First, this was a pilot study with a small sample size selected from a single center. Therefore, the metabolomic, proteomic, and miRNA biomarkers that differentiate MM patients with and without CVAEs need to be further validated before they can be considered useful clinically. Nevertheless, these preliminary findings point to a plausible pathway that indicates the CFZ-CVAEs might be related to mitochondrial dysfunction. Second, since the plasma samples were obtained after the patients were treated with CFZ, the identified biomarkers have no predictive value. In order to identify clinically useful predictive biomarkers, pre-treatment samples need to be analyzed. However, the differences between patients with and without CVAEs provided essential insights into the mechanisms of CFZ-CVAEs.

In summary, in this pilot multi-omics integrative analysis, we observed the down-regulation of pyruvate and up-regulation of LDHB among patients with CFZ-CVAEs, suggesting the importance of pyruvate oxidation pathway and mitochondrial dysfunction in CFZ-CVAEs. Further investigation in a larger independent cohort is warranted to better understand the mechanisms of CFZ-CVAEs.

## Data Availability Statement

The data presented in the study are deposited in the ProteomeXchange Consortium via the PRIDE partner repository, accession number (PXD024281).

## Ethics Statement

The studies involving human participants were reviewed and approved by University of Florida Institutional Review Board. The patients/participants provided their written informed consent to participate in this study.

## Author Contributions

MT wrote the manuscript. MT, LC, YH, and YG performed the statistical analyses. TG performed the metabolomics analysis. SS assisted in the pathway analysis. CS, NW, and JM collected the patient samples. YG, RC, RB, MF, DD, LY, TL, KM, and CP provided the critical revision of the manuscript. YG secured the funding for this study. All authors contributed to the article and approved the submitted version.

## Conflict of Interest

The authors declare that the research was conducted in the absence of any commercial or financial relationships that could be construed as a potential conflict of interest.

## References

[1] Siegel RL Miller KD Jemal A. Cancer statistics, 2019 (US statistics). CA Cancer J Clin. (2019) 69:7–34. 10.3322/caac.21551 30620402

[2] Gandolfi S Laubach JP Hideshima T Chauhan D Anderson KC Richardson PG. The proteasome and proteasome inhibitors in multiple myeloma. Cancer Meta Rev. (2017) 36:561–84. 10.1007/s10555-017-9707-8 29196868

[3] Hasinoff BB Patel D Wu X. Molecular mechanisms of the cardiotoxicity of the proteasomal-targeted drugs bortezomib and carfilzomib. Cardiovasc Toxicol. (2017) 17:237–50. 10.1007/s12012-016-9378-7 27388042

[4] Efentakis P Kremastiotis G Varela A Nikolaou PE Papanagnou ED Davos CH. Molecular mechanisms of carfilzomib-induced cardiotoxicity in mice and the emerging cardioprotective role of metformin. Blood. (2019) 133:710–23. 10.1182/blood-2018-06-858415 30482794

[5] Teicher BA Tomaszewski JE. Competitive landscape report. Biochem Pharmacol. (2015) 96:1–9. 10.1016/j.bcp.2015.04.008 25935605

[6] Dimopoulos MA Goldschmidt H Niesvizky R Joshua D Chng WJ Oriol A. Carfilzomib or bortezomib in relapsed or refractory multiple myeloma (ENDEAVOR): an interim overall survival analysis of an open-label, randomised, phase 3 trial. Lancet Oncol. (2017) 18:1327–37. 10.1016/S1470-2045(17)30578-8 28843768

[7] Libby E Garcia D Quintana D Fekrazad MH Bauman J Ebaid A. Disease-specific survival for patients with multiple myeloma: significant improvements over time in all age groups. Leuk Lymphoma. (2014) 55:2850–7. 10.3109/10428194.2014.897700 24588734

[8] Kistler KD Kalman J Sahni G Murphy B Werther W Rajangam K. Incidence and risk of cardiac events in patients with previously treated multiple myeloma versus matched patients without multiple myeloma: an observational, retrospective, cohort study. Clin Lymphoma, Myeloma Leuk. (2017) 17:89–96.e3. 10.1016/j.clml.2016.11.009 28025038

[9] Atrash S Tullos A Panozzo S Bhutani M Van Rhee F Barlogie B. Cardiac complications in relapsed and refractory multiple myeloma patients treated with carfilzomib. Blood Cancer J. (2015) 5:e272. 10.1038/bcj.2014.93 25594159 PMC4314456

[10] Siegel D Martin T Nooka A Harvey RD Vij R Niesvizky R. Integrated safety profile of single-agent carfilzomib: experience from 526 patients enrolled in 4 phase II clinical studies. Haematologica. (2013) 98:1753–61. 10.3324/haematol.2013.089334 23935022 PMC3815177

[11] Heckmann MB Doroudgar S Katus HA Lehmann LH. Cardiovascular adverse events in multiple myeloma patients. J Thorac Dis. (2018) 10:S4296–305. 10.21037/jtd.2018.09.87 30701098 PMC6328391

[12] Muchtar E Gatt ME Rouvio O Ganzel C Chubar E Suriu C. Efficacy and safety of salvage therapy using carfilzomib for relapsed or refractory multiple myeloma patients: a multicentre retrospective observational study. Br J Haematol. (2016) 172:89–96. 10.1111/bjh.13799 26567759

[13] Danhof S Schreder M Rasche L Strifler S Einsele H Knop S. ‘Real-life’ experience of preapproval carfilzomib-based therapy in myeloma - analysis of cardiac toxicity and predisposing factors. Eur J Haematol. (2016) 97:25–32. 10.1111/ejh.12677 26331915

[14] Yu AF Yadav NU Lung BY Eaton AA Thaler HT Hudis CA. Trastuzumab interruption and treatment-induced cardiotoxicity in early HER2-positive breast cancer. Breast Cancer Res Treat. (2015) 149:489–95. 10.1007/s10549-014-3253-7 25552363 PMC4970316

[15] Chong J Wishart DS Xia J. Using metaboanalyst 4.0 for comprehensive and integrative metabolomics data analysis. Curr Protoc Bioinforma. (2019) 68:e86. 10.1002/cpbi.86 31756036

[16] Xia J Psychogios N Young N WD. MetaboAnalyst: a web server for metabolomic data analysis and interpretation. Nucleic Acids Res. (2009) W652–60. 10.1093/nar/gkp356 19429898 PMC2703878

[17] Andersen CL Jensen JL Ørntoft TF. Normalization of real-time quantitative reverse transcription-PCR data: a model-based variance estimation approach to identify genes suited for normalization, applied to bladder and colon cancer data sets. Cancer Res. (2004) 64:5245–50. 10.1158/0008-5472.CAN-04-0496 15289330

[18] Livak KJ Schmittgen TD. Analysis of relative gene expression data using real-time quantitative PCR and the 2-ΔΔCT method. Methods. (2001) 25:402–8. 10.1006/meth.2001.1262 11846609

[19] Woo JM Kim JW Song JW Blank L1M Park JB. Activation of the glutamic acid-dependent acid resistance system in escherichia coli BL21(DE3) leads to increase of the fatty acid biotransformation activity. PLoS ONE. (2016) 11:e0163265. 10.1371/journal.pone.0163265 27681369 PMC5040553

[20] Brooks GA Dubouchaud H Brown M Sicurello JP Eric Butz C. Role of mitochondrial lactate dehydrogenase and lactate oxidation in the intracellular lactate shuttle. Proc Natl Acad Sci USA. (1999) 96:1129–34. 10.1073/pnas.96.3.1129 9927705 PMC15362

[21] Gewirtz DA. A critical evaluation of the mechanisms of action proposed for the antitumor effects of the anthracycline antibiotics adriamycin and daunorubicin. Biochem Pharmacol. (1999) 57:727–41. 10.1016/S0006-2952(98)00307-4 10075079

[22] Angsutararux P Luanpitpong S Issaragrisil S. Chemotherapy-induced cardiotoxicity : overview of the roles of oxidative stress. (2015) 2015:795602. 10.1155/2015/795602 26491536 PMC4602327

[23] Lipshultz SE Alvarez JA Scully RE. Anthracycline associated cardiotoxicity in survivors of childhood cancer. Heart. (2007) 94:525–33. 10.1136/hrt.2007.136093 18347383

[24] Comte B Vincent G Bouchard B Des Rosiers C. Probing the origin of acetyl-CoA and oxaloacetate entering the citric acid cycle from the 13C labeling of citrate released by perfused rat hearts. J Biol Chem. (1997) 272:26117–24. 10.1074/jbc.272.42.26117 9334176

[25] Herzig S Raemy E Montessuit S Veuthey JL Zamboni N Westermann B. Identification and functional expression of the mitochondrial pyruvate carrier. Science. (2012) 336:93–6. 10.1126/science.1218530 22628554

[26] Feng H Wu J Chen P Wang J Deng Y Zhu G. MicroRNA-375-3p inhibitor suppresses angiotensin II-induced cardiomyocyte hypertrophy by promoting lactate dehydrogenase B expression. J Cell Physiol. (2019) 234:14198–209. 10.1002/jcp.28116 30618075 PMC13483079

[27] Frey N Katus HA Olson EN Hill JA. Hypertrophy of the heart: a new therapeutic target? Circulation. (2004) 109:1580–9. 10.1161/01.CIR.0000120390.68287.BB 15066961

[28] Shimizu I Minamino T. Physiological and pathological cardiac hypertrophy [Internet]. J Mol Cell Cardiol. (2016) 97:245–62. 10.1016/j.yjmcc.2016.06.001 27262674

[29] Marcinkiewicz-Siemion M Ciborowski M Ptaszynska-Kopczynska K Szpakowicz A Lisowska A Jasiewicz M. LC–MS-based serum fingerprinting reveals significant dysregulation of phospholipids in chronic heart failure. J Pharm Biomed Anal. (2018) 154:354–63. 10.1016/j.jpba.2018.03.027 29571133

[30] Hoeflich A David R Hjortebjerg R. Current IGFBP-related biomarker research in cardiovascular disease-We need more structural and functional information in clinical studies. Front Endocrinol. (2018) 9:388. 10.3389/fendo.2018.00388 30061864 PMC6054974

[31] Di YF Li DC Shen YQ Wang CL Zhang DY Shang AQ. MiR-146b protects cardiomyocytes injury in myocardial ischemia/reperfusion by targeting Smad4. Am J Transl Res. (2017) 9:656–63. 28337293 PMC5340700

[32] Huynh K Barlow CK Jayawardana KS Weir JM Mellett NA Cinel M. High-Throughput plasma lipidomics: detailed mapping of the associations with cardiometabolic risk factors. Cell Chem Biol. (2019) 26:71–84.e4. 10.1016/j.chembiol.2018.10.008 30415965

[33] Zhu Y Feng Y Shen L Xu D Wang B Ruan K. Effect of metformin on the urinary metabolites of diet-induced-obese mice studied by ultra performance liquid chromatography coupled to time-of-flight mass spectrometry (UPLC-TOF/MS). J Chromatogr B Anal Technol Biomed Life Sci. (2013) 925:110–6. 10.1016/j.jchromb.2013.02.040 23523884

[34] Agarwal V Bell GW Nam JW Bartel DP. Predicting effective microRNA target sites in mammalian mRNAs. Elife. (2015) 4:1–38. 10.7554/eLife.05005.028 26267216 PMC4532895

[35] Shahini N Michelsen AE Nilsson PH Ekholt K Gullestad L Broch K. The alternative complement pathway is dysregulated in patients with chronic heart failure. Sci Rep. (2017) 7:1–10. 10.1038/srep42532 28195242 PMC5307342

[36] Berry M Galinier M Delmas C Fournier P Desmoulin F Turkieh A. Proteomics analysis reveals IGFBP2 as a candidate diagnostic biomarker for heart failure. IJC Metab Endocr. (2015) 6:5–12. 10.1016/j.ijcme.2014.11.003

[37] Barutaut M Fournier P Peacock WF Evaristi MF Caubère C Turkieh A. Insulin-like growth factor binding protein 2 predicts mortality risk in heart failure. Int J Cardiol. (2020) 300:245–51. 10.1016/j.ijcard.2019.09.032 31806281

[38] Li JW He SY Feng ZZ Zhao L Jia WK Liu P. MicroRNA-146b inhibition augments hypoxia-induced cardiomyocyte apoptosis. Mol Med Rep. (2015) 12:6903–10. 10.3892/mmr.2015.4333 26397753

[39] Fillmore N Mori J Lopaschuk GD. Mitochondrial fatty acid oxidation alterations in heart failure, ischaemic heart disease and diabetic cardiomyopathy. Br J Pharmacol. (2014) 171:2080–90. 10.1111/bph.12475 24147975 PMC3976623

[40] Lionetti V Stanley WC Recchia FA. Modulating fatty acid oxidation in heart failure. Cardiovasc Res. (2011) 90:202–9. 10.1093/cvr/cvr038 21289012 PMC3078800

[41] Li X Liu J Lu Q Ren D Sun X Rousselle T. AMPK: A therapeutic target of heart failure—not only metabolism regulation. Biosci Rep. (2019) 39:1–13. 10.1042/BSR20181767 30514824 PMC6328861

[42] Barry WH. Heart physiology from cell to circulation, 4th ed. Circulation. (2004) 110:e313. 10.1161/01.CIR.0000143724.99618.62

[43] Stanley W. Changes in cardiac metabolism: a critical step from stable angina to ischaemic cardiomyopathy. Eur Hear J Suppl. (2001) 3:02–7. 10.1016/S1520-765X(01)90147-6

