# Supplementary material for: Lactate Dehydrogenase B and Pyruvate Oxidation Pathway Associated With Carfilzomib-Related Cardiotoxicity in Multiple Myeloma Patients: Result of a Multi-Omics Integrative Analysis
Source: Front Cardiovasc Med. 2021 Apr 29;8:645122. doi: 10.3389/fcvm.2021.645122 (PMC8116486; doi:10.3389/fcvm.2021.645122)
Supplement: Supplementary file 1 [file Data_Sheet_1.docx]

**Supplemental Materials**

**Methods**

**Global Metabolomic Profiling**

All samples were analyzed in positive and negative heated electrospray ionization with a mass resolution of 35,000 at m/z 200 as separate injections. Separation was achieved on an ACE 18-pfp 100 x 2.1 mm, 2 µm column with mobile phase A as 0.1% formic acid in water and mobile phase B as acetonitrile. The flow rate was 350 µL/min with a column temperature of 25°C. 4 µL was injected for negative ions and 2 µL for positive ions.

Metabolites with > 50% missing were removed from further analyses. The remaining missing values of the endogenous compounds were imputed based on half of the minimum positive value in the original data, while missing values of metabolites of exogenous compounds were replaced with 0. Then the metabolite abundance values were normalized by sum, log-transformed and auto-scaled (mean-centered and divided by the standard deviation of each variable). Partial least squares discrimination analysis (PLS-DA) and t tests were performed to identify top metabolites that differentiate patients who developed carfilzomib-related HF versus those who did not.

**Global Proteomics Profiling**

Effective removal of high abundance proteins typically depends upon immunodepletion methods. We used the Seppro IgY14 column (Sigma-Adrich, St. Louis, MO, USA), which is designed to remove the 14 most abundant proteins. This method removed 95% of abundant proteins found in human plasma. The plasma proteins were then depleted using a micro bead column with avian antibody (IgY)-antigen and specialized buffers for sample loading, washing and eluting. Proteins were dissolved in denaturant (0.1% SDS (w/v)) and dissolution buffer (0.5 M triethylammonium bicarbonate, pH 8.5) included in the iTRAQ 8-plex kit (AB sciex Inc., Foster City, CA, USA). For each sample, a total of 100 μg of protein were reduced, alkylated, trypsin-digested, and labeled according to the manufacturer’s instructions (Sciex Inc., Foster City, CA, USA).

Labeled peptides were desalted with C18-solid phase extraction and dissolved in strong cation exchange (SCX) solvent A (25% (v/v) acetonitrile, 10 mM ammonium formate, and 0.1% (v/v) formic acid, pH 2.8). The peptides were fractionated using an Agilent HPLC 1260 with a polysulfoethyl A column (2.1 × 100 mm, 5 µm, 300 Å; PolyLC, Columbia, MD, USA). The absorbance at 280 nm was monitored and a total of 18 fractions were collected. A hybrid quadrupole Orbitrap (Q Exactive Plus) MS system (Thermo Fisher Scientific, Bremen, Germany) was used with high energy collision dissociation (HCD) in each MS and MS/MS cycle. The MS system was interfaced with an automated Easy-nLC 1000 system (Thermo Fisher Scientific, Bremen, Germany). Full MS scans were acquired in the Orbitrap mass analyzer over m/z 400–2000 range with resolution 70,000 at 200 m/z. The top ten most intense peaks with charge state ≥ 2 were isolated (with 2 m/z isolation window) and fragmented in the high energy collision cell using a normalized collision energy of 28%. The maximum ion injection time for the survey scan and the MS/MS scans were 250 ms, and the ion target values were set to 3e6 and 1e6, respectively.

The raw MS/MS data files were processed by a thorough database searching approach considering biological modification and amino acid substitution against the Uniprot *Daphnia magna* database (downloaded on August 30, 2017; 160,262 entries) using the ProteinPilot v5.0 with the Fraglet and Taglet searches under Paragon^TM^ algorithm^14^. The following parameters were considered for all the searching: fixed modification of methylmethane thiosulfonate-labeled cysteine, fixed iTRAQ modification of amine groups in the N-terminus, lysine, and variable iTRAQ modifications of tyrosine. The false discovery rate at the peptide level was estimated with the integrated PSPEP tool in the ProteinPilot Software to be 1.0%.
